# Educational Chronotopes in the Digital Landscape of Higher Education: A Systematic Bibliometric Mapping of Research Trends, 2015–2025

**DOI:** 10.12688/f1000research.186901.1

**Published:** 2026-08-06

**Authors:** Joshua Jacobs, Heloise Sathorar

**Affiliations:** 1 Nelson Mandela University Faculty of Education, Port Elizabeth, Eastern Cape, South Africa

**Keywords:** Chronotope; Critical Pedagogy; Digital Learning Environments; Higher Education; Teacher Education; Educational Space and Time; Bibliometric Analysis

## Abstract

**Background:**

The rapid expansion of digital learning in higher education has fundamentally altered the spatial and temporal dimensions, or chronotopes, of teaching and learning. This study maps the conceptual and intellectual landscape of educational chronotopes, exploring how critical pedagogy intersects with these shifting timespaces.

**Methods:**

This study employed a quantitative bibliometric research design to examine publication trends and the intellectual structure of the literature. Bibliometric indicators, including citation and keyword co-occurrence analyses, were generated in VOSviewer to identify influential publications, thematic clusters, and research networks within Higher Education.

**Results:**

The analysis identified four main paths shaping research on space and time in digital higher education: the integration of physical and virtual learning spaces into postdigital ecologies; pandemic-driven disruptions; the use of mobile and immersive tech to overcome spatial limits; and psychosocial and cultural factors supporting e-learning. Findings revealed a convergence between critical pedagogy and chronotopic views, emphasising intentional design of equitable digital environments that foster student agency and social interaction. However, the bibliometric network showed fragmentation among research themes, especially between emergency remote teaching, AI-driven systems, and critical pedagogy, highlighting the need for more integrated and sustainable digital education.

**Conclusion:**

Furthermore, the analysis demonstrates that critical pedagogy greatly informs chronotopic design by advocating for equitable, decentralised digital spaces that validate student voice. Finally, the study identifies significant fragmentation within the literature, particularly between quantitative research on AI-driven e-learning systems and postdigital pedagogies, and between reactive pandemic studies and proactive instructional design. To navigate future educational transformations, this study recommends bridging these structural gaps to design sustainable, highly engaging digital learning environments.

## 1. Introduction

Digital learning in higher education was significantly accelerated by the COVID-19 pandemic of 2020, which redefined teacher education by merging physical and virtual spaces into dynamic, hybrid environments. ^1^ By 2025, digital learning, encompassing both in-person and digital interactions via platforms such as learning management systems (LMS) and collaborative tools, promises a future of flexibility and inclusivity. ^2^ Research underscores the particular importance of digital learning during global disruptions, highlighting the urgent need for robust digital infrastructure and innovative pedagogies. ^3^

Information and Communications Technology (ICT) serves as the practical anchor for this knowledge-based transformation. However, for this transformation to be effective, ICT must be generalised across the education system, requiring fundamental reform that involves teachers as active pedagogical actors and stakeholders. Despite its advantages, ICT integration remains a multidimensional challenge; in teacher education, adoption is not merely a technical hurdle but a process relying on rational communication to guide necessary pedagogical adjustments.

Importantly, technology is not a neutral tool or force; rather, it acts as a mediating force that can either perpetuate social inequalities or foster emancipatory teaching practices. This is most evident in the “digital divide,” a multifaceted gap that represents a core social justice theme in educational technology. This divide encompasses disparities in connectivity, technological skills, and the capacity for knowledge creation among diverse socioeconomic groups. ^4^ Students with limited prior exposure to technology often face significant challenges when first introduced to these computing environments. ^5^

To analyse these complex interactions, this article utilises Bakhtin’s ^6^ construct of the chronotope, which refers to the “intrinsic connectedness of temporal and spatial relationships” (p. 84). In the digital realm, space is viewed as a relational, cultural, and social construct in which dialogue occurs, while time concerns the rhythms, pacing, and sequencing of education. ^7^ ^,^ ^8^ These hybrid environments function as dynamic sites of meaning-making, fundamentally shaping how lecturers in higher education institutions experience spatial presence and relational engagement. ^9^ Consequently, lecturers should design their learning environments to incorporate active learning through dialogue and sustained collaboration to navigate these timespaces effectively.

### 1.2 Theoretical framework

Against this backdrop of rapidly evolving digital timespaces and persistent inequalities, a critical pedagogical lens becomes essential for framing the significance of this inquiry. Within this perspective, notions of voice and participation are not peripheral but foundational to understanding how digital learning environments shape students’ identities. ^10^

Chronotopic perspectives, concerned with the interplay of space and time, intersect meaningfully with Critical Pedagogy by foregrounding the importance of context, interaction, and power in shaping educational experiences. As such, the post-digital paradigm should be understood not as neutral infrastructure but as socially constructed environments that require deliberate, ethical design. This involves fostering the mutual co-construction of knowledge within shared problem spaces, ^11^ where students and lecturers engage in dialogical negotiation of meaning. ^12^ Consequently, this study argues that revisiting the chronotope of digital education is important for promoting equity, social interaction, and transformative learning. However, despite this recognition, the field remains fragmented and lacks a systematic synthesis of how these dynamics are conceptualised and applied, highlighting the need to identify a clear research gap.

## 2. Identifying the research gap

The landscape of higher education research has seen a significant surge in studies on digital learning and hybrid teaching, particularly in response to the disruptions caused by the COVID-19 pandemic. ^1^ While current literature extensively covers themes such as ICT integration, teacher competencies, and student engagement, ^13^ ^,^ ^2^ these discussions often focus on technology as a tool rather than exploring the fundamental “timespace” shifts it creates. Although Bakhtin’s ^6^ concept of the chronotope has been introduced as a theoretical lens for understanding the “intrinsic connectedness of temporal and spatial relationships” in education, its application remains largely fragmented. ^8^

A critical gap exists in the systematic synthesis of this field. While qualitative research has explored specific temporal dimensions and dialogic sense-making, ^14^ ^,^ ^15^ there is a notable absence of research that maps the intellectual and conceptual landscape of chronotopes across the broader discipline. Conventional literature reviews often fall short because they rely on manual selection and are prone to sample selection bias, making them inadequate for navigating the “information explosion” of recent scholarly output. ^16^

To date, no comprehensive bibliometric mapping has been conducted to quantitatively visualise the thematic clusters that define chronotopic research in digital learning. Consequently, the experience space and time in computer-mediated environments remain underexplored and unmapped. ^17^ ^,^ ^18^ Without a data-driven mapping of these trends, scholars lack a clear framework to identify underexplored areas or to understand how chronotopic design can effectively bridge the digital divide and foster social justice in a post-pandemic era. ^4^

### 2.1 Guiding research questions

This study applied the bibliometric analysis methodology presented by Caputo et al. ^19^ to create a knowledge framework of chronotopic research in digital learning. A bibliometric analysis of the past decade maps the conceptual and intellectual landscape of chronotopes in digital learning. The aim of this review is to guide Higher Education practitioners in designing empowering, dialogic, and equitable computer-mediated learning spaces. ^14^ ^,^ ^9^

By conducting a bibliometric analysis, the study identifies the breadth of existing scholarship, recent developments, and persistent gaps within the evolving landscape of higher education. It mapped key trends and emerging areas of inquiry while highlighting the real-world implications and unresolved challenges that warrant further exploration. In doing so, the review uncovered theoretical trajectories and global perspectives that have shaped the discourse on digital learning to improve human-computer interaction.

RQ1: What are the main conceptual and thematic trends shaping the discourse on space and time in digital education in Higher Education (or in teacher education)?

RQ2: How does critical pedagogy inform or intersect with chronotopic perspectives identified in the review on digital learning?

RQ3: What emerging themes and research gaps can be identified for future studies on chronotopes?

## 3. Methodology

This review follows the PRISMA 2020 guidelines ^20^ to examine how existing scholarship has used the chronotope as a theoretical lens in studies of digital learning in higher and teacher education. The review process was designed to ensure transparency, replicability, and a systematic approach to identifying, screening, and analysing the relevant literature (see Figure 1).

**Figure 1.**
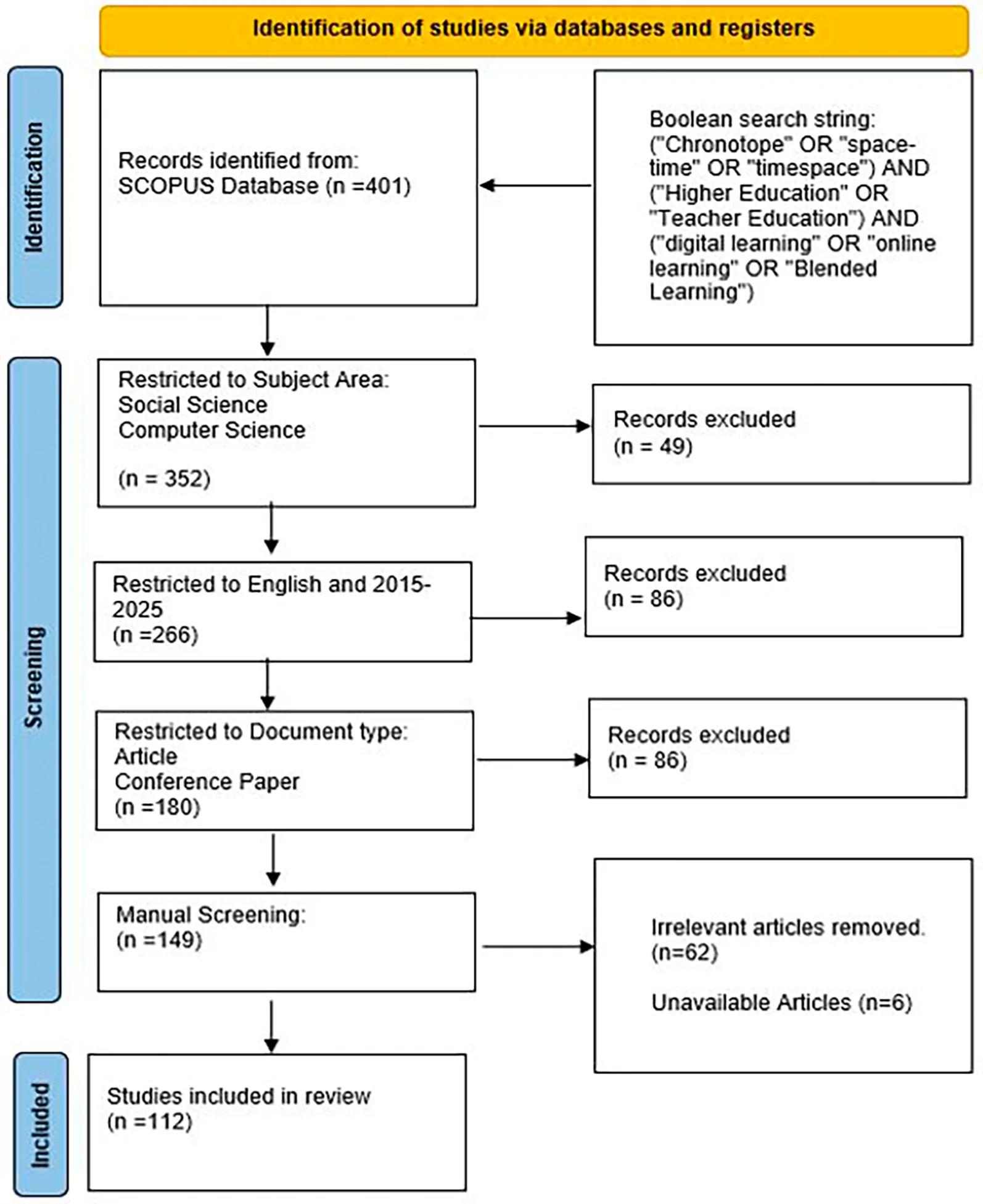
PRISMA Diagram.

### 3.1 Identification

In this review, the chronotope is defined operationally as a dialogical and analytical framework for understanding how the spatial and temporal dimensions are socially constructed, entangled, and dynamically negotiated by participants in technology-mediated educational settings. ^6^ ^,^ ^21^ This definition informed the search strategy and inclusion/exclusion criteria, ensuring that the review captured studies addressing the complex spatial-temporal dynamics of contemporary educational paradigms. For example, shifting educational chronotopes manifest in blended-learning course architectures that require the cohesive integration of physical and virtual interactions. ^22^ In postdigital learning ecosystems where digital tools actively blur traditional pedagogical boundaries and hierarchies ^23^ ^,^ ^24^ and in emergency online (im) mobility chronotopes that paradoxically compress social, domestic, and academic spaces ^25^ ^,^ ^26^ While these diverse applications are grouped under the broad labels of digital or blended learning, they pose qualitatively different pedagogical dilemmas for educators striving to design equitable, supportive, and meaningful social spaces.

To refine the scope of this review, we focused on literature exploring the intersection of these spatial-temporal phenomena with modern digital pedagogies. The primary database utilised for data collection was Scopus, selected for its robust, high-quality coverage of interdisciplinary research spanning educational technology, higher education, and the social sciences. The search strategy targeted publications that explicitly addressed the theoretical and practical restructuring of learning environments. Accordingly, the following Boolean search string was adapted and applied to the database:

(“Chronotope” OR “space-time” OR “timespace”) AND (“Higher Education” OR “Teacher Education”) AND (“digital learning” OR “online learning” OR “Blended Learning”).

This initial search yielded 401 records from the Scopus database on the 4th of March 2026. Following the retrieval, a systematic screening process was conducted to assess data quality, remove duplicates, and ensure thematic relevance. The identified records were subjected to rigorous inclusion and exclusion criteria (see Figure 1), limiting the final dataset to peer-reviewed articles that provided empirical or robust theoretical insights into the operationalised concepts of the chronotope or space-time within tertiary and teacher education.

### 3.2 Screening

Following the initial retrieval of 401 records from the Scopus database, a multi-stage systematic screening process was conducted in accordance with the Preferred Reporting Items for Systematic Reviews and Meta-Analyses (PRISMA) guidelines to ensure data quality and thematic relevance. ^27^ In the first stage of screening, the dataset was restricted to the subject areas of Social Science and Computer Science, resulting in the exclusion of 49 records and the retention of 352.

To ensure analytical depth and focus on contemporary digital learning paradigms, temporal and linguistic parameters were subsequently applied, limiting the results to English-language publications between 2015 and 2025. This removed 86 records, leaving 266 for further evaluation.

The scope was then narrowed by document type to include only peer-reviewed articles and conference papers, thereby excluding 86 records, such as book chapters, as full-text access for book chapters is restricted to external publisher sites, editorial materials, or reviews, yielding a refined dataset of 180 publications.

A rigorous manual screening of the remaining records was conducted to assess their relevance to the study, see Figure 1, using predefined inclusion and exclusion criteria. ^28^ During this stage, each article’s title, abstract, and, where necessary, the full text, were carefully reviewed to determine alignment with the study’s operationalised concepts. From the 149 records that underwent manual screening, 62 articles were excluded because they were not sufficiently relevant to the focus of the review. A further 6 records were removed due to the unavailability of the full text. This process yielded a final corpus of 112 articles that met the inclusion criteria for the review. These articles were subsequently exported for bibliometric analysis using VOSviewer.

### 3.3 Data extraction and synthesis

Following the screening process, a final dataset of 112 articles was retained for analysis. Bibliographic data from these studies were exported and analysed using VOSviewer, a specialised software tool designed for constructing and visualising bibliometric networks. ^29^ The software enabled the examination of relationships within the literature through several complementary bibliometric techniques, including citation analysis, bibliographic coupling, co-citation analysis, and keyword co-occurrence analysis.

Citation analysis was used to identify influential publications in the dataset by examining how often each article was cited by other studies, thereby indicating its intellectual impact in the field. ^30^ Bibliographic coupling was employed to detect relationships between publications based on shared references, operating on the premise that studies citing the same sources are likely to address related topics. ^31^ ^,^ ^19^ Co-citation analysis further examined the intellectual structure of the field by identifying publications or authors that are frequently cited together, thereby revealing conceptual linkages and research traditions within the literature. ^19^

To explore the field’s conceptual structure, a keyword co-occurrence analysis (co-word analysis) was conducted using author-supplied keywords. This technique identifies frequently co-occurring terms in the literature and allows for the detection of thematic groupings that represent dominant research topics. ^32^ ^,^ ^19^ VOSviewer generated network and density visualisations, grouping keywords into clusters based on their co-occurrence patterns (See Figure 3). In these visualisations, the size of each node represents the frequency of occurrence, while the distance between nodes reflects the strength of their relationship. Clusters are represented by different colours, indicating distinct thematic streams within the research field.

The resulting visual network maps revealed key thematic clusters, influential research trajectories, and the relationships between emerging areas of scholarship. These clusters were subsequently interpreted qualitatively to understand how the literature conceptualises the interaction between space and time in digital learning environments. In this way, the bibliometric analysis complemented the systematic review by providing a quantitative mapping of the field, enabling the identification of dominant themes, influential works, and gaps within the chronotopic discourse on digital learning.

## 4. Presentation of data

This section presents the results of the bibliometric analysis, including publication trends from 2015–2025 and the thematic clusters identified through network, density, and overlay visualisations ( Figures 3, 4, and 5).

### 4.1 Publication trends from 2015 to 2025

Figure 2 illustrates the annual publication trends from 2015 to 2025, revealing a distinct upward trajectory in scholarship and indicating rapidly expanding academic interest in the research domain. During the initial period from 2015 to 2017, research output remained static at a baseline of five articles per annum, followed by a notable contraction to a single publication in 2018. However, a substantial resurgence began in 2019, with 8 articles, establishing a foundation for an accelerated growth phase that closely aligned with the onset of the global health crisis. The publication volume rose steadily from 10 articles in 2020 to 13 in 2021, then reached an initial peak of 17 in 2022.

**Figure 2.**
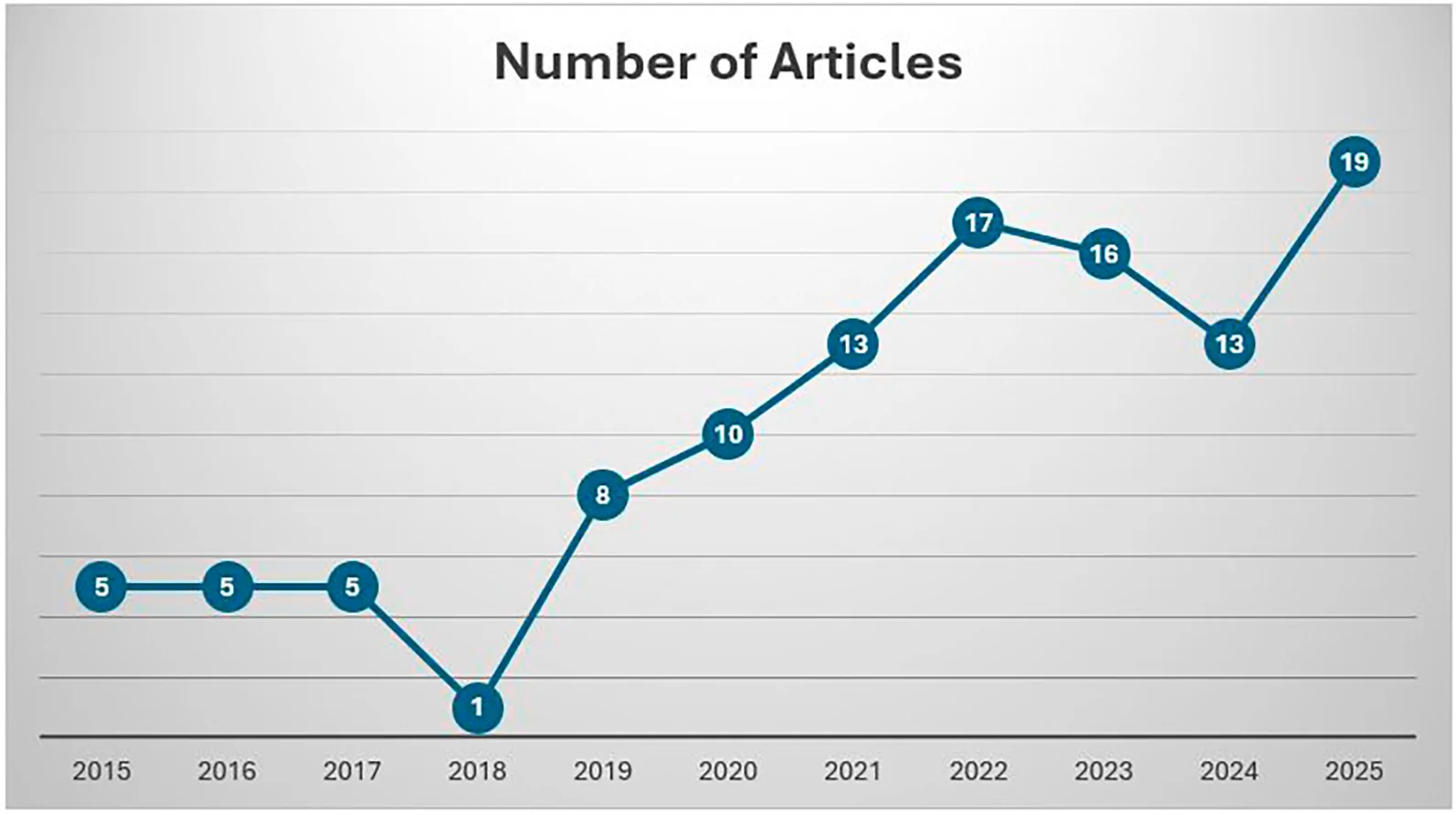
Articles per year.

Contextualising this data within the broader discourse on digital learning and educational chronotopes, this significant escalation during the pandemic years can be directly attributed to the abrupt global transition to emergency remote teaching. The forced shift to online modalities necessitated urgent scholarly inquiry into the profound spatiotemporal disruptions in higher education institutions, prompting researchers to critically examine their impact on student equity, well-being, and engagement. Although there was a minor regression in output to 16 articles in 2023 and 13 articles in 2024, the overarching upward trend ultimately culminated in an unprecedented peak of 19 publications in 2025. This sustained and amplified proliferation of literature by 2025 reflects a transition towards a mature, postdigital paradigm in higher education.

Table 1 shows the most-cited publications, highlighting the focus on learning spaces, student experiences, online and campus teaching, and digital technologies. These works suggest digital learning is viewed as a socio-spatial phenomenon shaped by interactions among learners, technologies, contexts, and practices.

**Table 1.** Top 10 Cited Articles.

	Top cited publications	Author(s)	Cited	Type of Study	Method	Data Source	Keywords
**1.**	Grit in the path to e-learning success	Aparicio, M., Bacao, F., & Oliveira, T.	243	Quantitative empirical study	Structural Equation Modelling (SEM) utilising the Partial Least Squares (PLS) method.	A survey was administered to 383 university students.	e-learning success; e-learning systems; Grit; Perseverance; Stamina; Success determinants
**2.**	Models of learning space: integrating research on space, place and learning in higher education	Ellis, R. A., & Goodyear, P.	214	Conceptual paper/Literature review	Qualitative synthesis and review of dispersed and fragmented literature across multiple domains.	Relevant higher education learning space literature, predominantly published from 1995 onwards.	higher education; Learning space research; physical and virtual learning spaces; place and learning; space
**3.**	Cultural impacts on e-learning systems’ success	Aparicio, M., Bacao, F., & Oliveira, T.	156	Quantitative empirical study	Structural Equation Modeling (SEM) assessed via Partial Least Squares (PLS) algorithm.	An online electronic survey yielding 323 valid responses from students across 11 higher education institutions.	Culture; e-Learning systems; Individualism/collectivism; Satisfaction; Success model; Use
**4.**	The covid-19 pandemic as an opportunity to foster the sustainable development of teaching in higher education	Sá, M. J., & Serpa, S.	150	Perspective paper/Qualitative research	Content analysis and documentary analysis utilizing cumulative screening stages.	Articles, books, and book chapters retrieved from B-ON and SCILIT databases.	COVID-19; Digital sustainable development; Digital transformation; Higher education; Leadership; Sustainability; Teaching; University performance
**5.**	Learning to be a writer: A spherical video-based virtual reality approach to supporting descriptive article writing in high school Chinese courses	Huang, H. L., Hwang, G. J., & Chang, C. Y.	127	Quasi-experimental study,	Mixed methods, employing ANCOVA for quantitative test evaluation and grounded theory for qualitative focus-group interviews.	65 11th-grade senior high school students (30 experimental, 35 control) in Taiwan.	No Keywords Listed
6.	An exploration of students’ lived experiences of using smartphones in diverse learning contexts using a hermeneutic phenomenological approach	Chan, N. N., Walker, C., & Gleaves, A. (2015).	77	Qualitative phenomenological study,	Hermeneutic phenomenological approach utilizing semi-structured interviews and written reflective exercises.	12 youths (aged 16–19) from secondary schools and tertiary colleges in Malaysia.	Lived experiences; Mobile learning; Phenomenology; Smartphones; Students
7.	Reading and connecting: using social annotation in online classes	Zhu, X., Chen, B., Avadhanam, R. M., Shui, H., & Zhang, R. Z.	73	Case study	Mixed methodology involving the collection and analysis of both quantitative and qualitative data.	An online class treated as the focal case.	Collaborative learning; Collaborative reading; Online learning; Social annotation; Social reading; Web annotation
8.	A Learning Experience in Inquiry-Based Physics with Immersive Virtual Reality: Student Perceptions and an Interaction Effect Between Conceptual Gains and Attitudinal Profiles	Tsivitanidou, O. E., Georgiou, Y., & Ioannou, A. (2021).	58	Quantitative study	Statistical analysis of Likert-style scale responses adapted from the Test of Science-Related Attitudes (TOSRA).	Pre-surveys, pre-post conceptual tests, and post-activity questionnaires completed by students.	Digital technologies attitudes; Immersive virtual reality (VR); Learning experience; Physics education; Scientific attitudes; Technology integration
9.	International student mobilities in a contagion: (Im) mobilising higher education?	Sidhu, R., Cheng, Y. E., Collins, F., Ho, K. C., & Yeoh, B.	50	Conceptual analysis/Literature review,	Theoretical and contextual analysis of digital infrastructure and policy trends.	Academic literature and reports on technology-mediated learning and educational disruption.	digital infrastructure; higher education; international student mobilities; market making; subject making
10.	Dissolving the Dichotomies Between Online and Campus-Based Teaching: a Collective Response to The Manifesto for Teaching Online (Bayne et al. 2020)	MacKenzie A, Bacalja A, Annamali D, Panaretou A, Girme P, Cutajar M, Abegglen S, Evens M, Neuhaus F, Wilson K, Psarikidou K	39	Collective response/Conceptual reflection	Dialogic reflection and collaborative theoretical synthesis.	Contributions, literature, and reflections from academic practitioners in postdigital science and education.	Campus learning; Collective response; Covid-19; Digital learning; Distant learning; Manifesto for teaching online; Postdigital.

### 4.2 Cluster 1: AI-Driven E-Learning systems and mobile learning ecosystems

This Red cluster of 21 keywords underscores the pervasive role of digital platforms in contemporary education, with a predominant focus on “e-learning,” “students,” and “learning systems”. The prominence of these high-weight keywords suggests that the core of this research cluster revolves around the adoption, success, and user satisfaction of e-learning systems in supporting student “academic performance” ^33^ ^,^ ^34^ Secondary themes are highlighted by keywords such as “education computing” and “artificial intelligence,” indicating a paradigm shift toward data-driven, “personalized learning” environments that enhance instructional “efficiency” and optimize educational outcomes. ^35^ ^,^ ^36^ Furthermore, keywords with specific contextual importance, such as “mobile learning,” “smartphones,” “smartphone,” “information use,” and “social networking (online),” illustrate the importance of ubiquitous technologies and “social media” that allow students to seamlessly access information across formal and informal spaces. ^37^

Interestingly, the inclusion of the keyword “japan” alongside inquiries into mobile devices reflects the global scope of research, with its presence largely attributable to its frequency within the co-occurrence patterns of the reviewed literature. The cluster also reveals a focus on pedagogical structuring through keywords like “virtual reality,” “learning objects,” “curricula,” and “teaching modes,” suggesting efforts to redesign educational delivery utilizing immersive virtual environments and dynamic digital materials. ^38^ ^,^ ^39^

Finally, the presence of “professional development” and “quality control” highlights the necessity of equipping educators with ongoing training to effectively integrate these advanced technologies into their pedagogical practices while maintaining high standards of educational delivery. ^35^ ^,^ ^40^ ^,^ ^41^ Building on the analysis from this cluster, it will be helpful to conduct further research into how artificial intelligence, immersive technologies, and mobile social networking can be effectively combined to create highly personalized, efficient e-learning systems that continuously improve student performance and educator development.

### 4.3 Cluster 2: Chronotopic design of blended learning in higher education

This thematic cluster, represented in the green region with 13 keywords, underscores the intricate entanglement of space, time, and technology in contemporary educational paradigms. The cluster centres on the transformation of “higher education” and “teaching” within hybrid environments, as evidenced by keywords such as “blended learning,” “educational technology,” and “computer aided instruction”. Higher education is increasingly characterised by the integration of online and face-to-face modalities, requiring a redesign of learning spaces that merge physical and virtual interactions. The keywords “chronotope” and “social space” suggest a critical focus on the spatial-temporal dimensions of these environments. The dialogical notion of the chronotope serves as an analytical tool to understand how space and time are socially constructed, entangled, and dynamically negotiated by participants during collaborative “teaching activities”. ^8^ Furthermore, the prominence of “design” and “pedagogy” underscores the need to rethink course architectures to move beyond a simplistic online/offline divide. ^22^ This highlights a strong theme of aligning technological affordances with constructivist pedagogical principles, ensuring that educational technology serves as an environment for meaningful social interaction rather than merely as a vehicle for information delivery. The integration of the “internet” and “digital citizenship” points to a broader concern about how networked platforms shape learners’ identities, emphasising the need to prepare students to contribute positively to society through technology. ^10^ Additionally, the presence of “qualitative research” highlights the methodological approaches employed to explore these multifaceted phenomena. Qualitative methods, such as narrative inquiry, are frequently utilised to capture the nuanced, lived experiences, meaning-making processes, and spatial-temporal navigations of students and educators in technology-mediated environments. ^42^ Building on the analysis from this cluster, future research could delve deeper into how educators can effectively design blended learning spaces that foster equitable social interactions and robust digital citizenship, exploring how shifting educational chronotopes continuously redefine the boundaries of teaching and learning.

### 4.4 Cluster 3: Psychological determinants of E-Learning in STEM education

Represented by the Blue cluster of 13 keywords. This cluster highlights the psychological dimensions and methodological approaches used to evaluate digital instruction, with a strong focus on technical disciplines. The prominence of keywords like “engineering education” and “STEM (science, technology, engineering and mathematics)” suggests a strong theme of leveraging technology to cultivate specialised competencies and concepts within technical fields. ^11^ This cluster seems to centre around the cognitive and affective states of the “human” learner interacting with “e-learning systems” and “e - learning,” as evidenced by keywords such as “motivation,” “self-efficacy,” and “satisfaction”. ^43^ ^,^ ^11^ These keywords highlight that, in online and blended environments, a learner’s self-efficacy and intrinsic motivation are critical drivers of high student satisfaction and deep conceptual understanding in STEM subjects. ^44^

Keywords such as “information literacy” and “skill” indicate a continued concern with equipping students with the evaluative proficiencies required to navigate complex online spaces, process massive amounts of data, and effectively acquire knowledge. ^45^ The presence of the keyword “teacher education” indicates a concern about how institutions are preparing educators to seamlessly integrate technology into their pedagogy, ensuring they can appropriately foster these essential psychological traits and digital skills in their students. ^43^ The presence of keywords like “least squares approximations” and “article” suggests that many studies within this cluster employ rigorous quantitative research methods—specifically Partial Least Squares Structural Equation Modeling (PLS-SEM)—to map the complex pathways between e-learning behaviours, technology acceptance, and academic performance. ^34^

Building on the analysis from this cluster, it will be helpful to conduct further research on how specific e-learning systems and digital tools can be optimally designed to boost motivation, mitigate need frustration, and enhance self-efficacy among STEM students. ^11^ Furthermore, research could examine the design and effectiveness of targeted teacher education programs that foster robust information literacy and equitable, highly satisfying learning experiences across diverse digital formats. ^45^

### 4.5 Cluster 4: Pandemic-Induced educational chronotopes and student mobility

Represented as a Yellow cluster with 12 keywords, this cluster highlights the profound spatiotemporal disruptions to transnational education. Most notably, focusing on how the global health crisis altered the lived realities of mobile learners. The prominence of keywords like “covid-19”, “student”, “pandemic”, and “time” suggests a strong theme exploring how the “epidemic” fundamentally suspended traditional educational pathways and rhythms. ^26^ Keywords such as “international students”, “international migration”, “migration determinant”, and “china” highlight a specific research emphasis on the stalled trajectories of learners—particularly Chinese international students navigating strict borders—who were forced to inhabit states of involuntary immobility. ^26^ Notably, the presence of keywords like “learning space”, “time”, and “learning experience” points toward the necessity of analyzing these phenomena through the theoretical lens of Bakhtinian chronotopes, which capture the intrinsic entanglement of spatial and temporal dimensions in education. ^46^ In the context of remote emergency teaching, the traditional physical educational chronotope was replaced by an “online (im) mobility chronotope,” in which students were physically grounded yet digitally transnational, creating a paradoxical compression of social, domestic, and academic spaces. ^25^ This dislocation generated asynchronous and precarious temporalities that severely impacted student “wellbeing” and the overall “learning experience”, as learners grappled with sticky, suspended times and the loss of expected life course progressions. ^8^ ^,^ ^26^ The blurring of private, domestic spaces with public, academic time zones forced students to inhabit liminal non-places, complicating their educational engagement and sense of belonging. ^25^ It will be helpful to conduct further research on how educators can design more equitable and supportive digital chronotopes that mitigate the temporal and spatial precarities faced by immobile transnational students. Additionally, future research can delve further into strategies to sustain students’ “wellbeing” and agency amid the unpredictable “migration determinant” factors shaping global education today. ^26^

### 4.6 Cluster 5: Postdigital social media and sustainable digital learning

Represented by this Purple cluster of nine keywords, this grouping underscores the evolving landscape of “digital learning” and “education” within contemporary, technology-mediated environments. The prominent frequency of “Social Media alongside “learning” suggests a strong focus on how social networking platforms are leveraged to facilitate interactive, decentralised, and student-led learning communities. ^47^ Within these environments, “Social Media” is no longer viewed merely as a passive delivery tool but as an active, connective space that promotes the co-production of knowledge and blurs traditional pedagogical hierarchies. ^47^ Furthermore, the emergence of the “postdigital” keyword reflects a paradigm shift in which digital technologies and social media are increasingly understood as inextricably intertwined with the everyday material and social practices of higher education. ^24^ This postdigital perspective rejects the binary separation of digital and physical domains, emphasising instead how digital tools are already embedded within the complex boundaries of learning environments. ^48^ ^,^ ^49^

Keywords such as "sustainability" and "sustainability education" underscore the imperative to align these "digital learning" transformations with value-driven, future-oriented goals. ^50^ ^,^ ^24^ Higher education institutions are increasingly tasked with redesigning "education" not only to integrate advanced digital learning platforms but also to equip students with the capabilities necessary to navigate global uncertainties and contribute to sustainable development. ^51^ Finally, the presence of "open education" and "voice” points toward efforts to democratize access and empower student expression, recognising that flexible and inclusive digital environments can mitigate barriers and foster a sense of belonging and active participation across diverse student populations. ^52^ ^,^ ^50^ Building on this cluster, future research should explore how the integration of social media and postdigital pedagogies can be optimised to create sustainable, equitable, and highly engaging digital learning ecosystems that continuously support student voice and collaborative knowledge construction. ^50^

### 4.7 Cluster 6: Collaborative engagement in online and virtual learning environments

Represented by this Cyan cluster of nine keywords, this grouping demonstrates the necessity of fostering meaningful “student engagement” within the complex, technology-mediated environments of contemporary “online education” and “remote learning”. ^53^ ^,^ ^54^ The cluster centres heavily on the deployment of a “virtual team” or “virtual teams” to facilitate active “collaboration” across geographical boundaries, emphasising that deep learning in digital spaces relies on dynamic peer-to-peer “knowledge sharing”. ^55^ ^,^ ^54^ Within this context, “collaboration” transcends simple group work; it requires intentional instructional design to support the social construction of knowledge and to prevent the isolation often associated with remote learning. ^57^ Furthermore, the presence of “digital literacy” highlights a crucial prerequisite for this meaningful engagement. Students must possess the necessary digital competencies not only to consume information but also to actively communicate, coordinate, and critically evaluate content within virtual team environments. ^55^ The prominence of “leadership” points to the vital role of both instructors and student leaders in orchestrating these virtual ecosystems. Effective “leadership” is required to establish psychological safety, manage diverse team dynamics, and scaffold the learning process, ensuring that technological tools serve pedagogical goals rather than dictate them. ^55^ ^,^ ^54^ Building on the analysis from this cluster, future research should explore how targeted digital literacy interventions and distributed leadership models can optimise virtual team collaboration, ultimately sustaining meaningful student engagement and high-quality knowledge sharing in post-pandemic online education.

### 4.8 Cluster 7: Gamified and game-based learning in online education

Represented by an orange cluster of six keywords, this grouping underscores the integration of interactive, gamified pedagogies to mitigate the spatiotemporal disruptions caused by the “COVID-19 pandemic” in higher education. The prominent frequencies of “online learning” and the “covid-19 pandemic” highlight a primary research focus on the abrupt, widespread transition to remote education and the ensuing challenges of maintaining student engagement, mitigating attrition, and supporting psychological well-being during periods of forced isolation. ^56^ ^,^ ^34^ To address these challenges, the cluster demonstrates a significant reliance on “game-based learning” and the deployment of an “educational game” or “computer games” to foster immersive, highly motivating digital environments. Game-based learning approaches are uniquely positioned to enhance behavioural, cognitive, and affective engagement by satisfying students’ basic psychological needs and facilitating optimal flow experiences, which counteract the disengagement often associated with traditional online learning tasks. ^11^

Furthermore, the presence of “collaborative learning” indicates that these technological interventions are deeply rooted in social constructivist frameworks. Rather than isolating learners, effectively designed online games and digital learning ecosystems require dynamic peer-to-peer interaction, structured role-taking, and the mutual co-construction of knowledge within a joint problem space. ^11^ Building on the analysis from this cluster, future research should explore how the specific affordances of computer games and structured collaborative learning scripts can be optimally integrated to create resilient, highly personalised online learning ecosystems that sustain student motivation and complex problem-solving skills well beyond the emergency remote teaching phase. ^56^

## 5. Discussion of findings

An analysis of the thematic coupling results reveals five key areas where critical pedagogy could intersect with contemporary digital learning paradigms. The intersection of critical pedagogical principles and chronotopic perspectives within digital learning primarily manifests in the conscious redesign of educational space and time to foster equity, student agency, and social interaction. The dialogical notion of the chronotope provides a crucial analytical framework for understanding how spatial and temporal dimensions are socially constructed, entangled, and dynamically negotiated by participants during collaborative educational activities. ^6^ ^,^ ^12^

### 5.1 The Democratisation of knowledge and blurring of pedagogical hierarchies

Critical pedagogy seeks to dismantle oppressive educational structures by empowering student voice and agency. This objective aligns directly with the emergence of postdigital social media ecosystems (Cluster 5), wherein digital platforms are leveraged to facilitate interactive, decentralised, and student-led learning communities. ^48^ Within this paradigm, social media transcends its function as a passive delivery tool and becomes an active, connective space that promotes the co-production of knowledge, thereby fundamentally blurring traditional pedagogical hierarchies. ^48^ Consequently, the integration of social media within educational contexts not only facilitates the democratisation of knowledge but also actively reconfigures traditional power relations, positioning learners as co-constructors rather than passive recipients of knowledge, a process further supported by gamified pedagogies (Cluster 7) that provide interactive joint problem spaces where students exercise agency and actively dismantle traditional pedagogical hierarchies.

### 5.2 Designing equitable digital chronotopes to mitigate educational uncertainty

The intersection of critical pedagogy and digital learning is greatly spatial and temporal, necessitating the design of equitable digital chronotopes (Cluster 2 and Cluster 4). The global health crisis exposed severe spatiotemporal disruptions, replacing the physical educational chronotope with an “online (im) mobility chronotope” that physically grounded students while forcing them to inhabit precarious, asynchronous liminal spaces. ^25^ ^,^ ^26^ This dislocation severely impacted student well-being and agency, highlighting an urgent need for educators to critically analyse these phenomena. ^46^ A critical pedagogical approach demands the conscious redesign of blended learning architectures to mitigate these spatial and temporal uncertainties from the lecturer’s perspective. The purpose of moving beyond simplistic course design is to foster environments that prioritise social interaction. ^22^

Addressing these spatiotemporal perspectives of lecturers’ critical pedagogical interventions to design more equitable and supportive digital chronotopes that actively mitigate the precarities faced by immobile or marginalised learners. Consequently, course design must be rethought to move beyond simplistic online-offline binaries and purposefully align technological affordances with constructivist pedagogical principles. By doing so, educational technology ceases to function merely as a vehicle for passive information delivery and instead becomes a conduit for meaningful social interaction, epistemic access, and the empowerment of student expression. This critical approach should aim to facilitate student-led communities oriented toward the co-production of knowledge. ^23^

### 5.3 Aligning digital transformation with sustainable and value-driven education

Critical pedagogy insists that education must be oriented toward broader societal transformation and justice. This aligns with the postdigital imperative to connect digital learning transformations to value-driven, future-oriented goals, such as sustainability education (Cluster 5). ^50^ ^,^ ^24^ Higher education institutions are increasingly tasked with redesigning curricula not merely to integrate advanced platforms or optimise instructional efficiency, but to equip students with the critical capabilities necessary to navigate global uncertainties and actively contribute to sustainable development. ^51^ This requires a postdigital perspective that recognises digital tools as inextricably embedded within the complex material and social boundaries of higher education. ^47^ ^,^ ^49^

The dialogic nature of critical pedagogy relies on mutual knowledge sharing and communal problem-solving, which must be carefully scaffolded in remote learning environments (Cluster 6). Meaningful student engagement in online education relies heavily on the deployment of virtual teams to facilitate active collaboration across geographical boundaries. ^53^ ^,^ ^54^ However, from a critical perspective, this collaboration must transcend simple group work; it requires intentional instructional design to support the social construction of knowledge and to prevent the isolation often associated with remote learning. ^57^ Furthermore, one could argue that you would also want epistemological growth among the students so that they can view society from varied perspectives. Effective, distributed leadership from the lecturer is therefore vital to establishing psychological safety, managing diverse team dynamics, and ensuring that technological tools serve pedagogical goals rather than dictate them. ^55^ ^,^ ^54^

In synthesis, critical pedagogy and chronotopic perspectives intersect by exposing the deeply political nature of space and time in digital education. By critically examining how educational time and space are socially constructed, regulated, and experienced, educators can dismantle oppressive hierarchies and design postdigital learning environments that foster genuine dialogic engagement, equity, and robust digital citizenship.

### 5.4 Prominent conceptual trends in top cited articles:

An analysis of the most highly cited literature reveals that the bibliometric discourse on space and time in digital higher education is characterised by four primary conceptual and thematic trends, ranging from the integration of virtual and physical environments to the spatiotemporal impacts of global disruptions.

The most prominent conceptual trend involves the structural reconceptualisation of learning environments. The second most cited publication in the dataset (214 citations) is a conceptual literature review that synthesises fragmented research from 1995 onwards to develop integrated models of “physical and virtual learning spaces,” emphasising the critical relationship between “place and learning” in higher education. This thematic trajectory toward spatial integration is further evidenced by collective reflections that aim at “Dissolving the Dichotomies Between Online and Campus-Based Teaching”. Garnering 39 citations, this dialogic synthesis leverages “Postdigital” frameworks to challenge the binary separation of “Campus learning” and “Distant learning.” A second major thematic trend centres on the systemic spatiotemporal disruptions caused by global crises, particularly the COVID-19 pandemic, and their effect on student mobility and institutional sustainability. Bibliometric data highlights a strong focus on spatial immobility and infrastructural adaptation, as seen in conceptual analyses of “International student mobilities in a contagion”. Concurrently, studies analysing the pandemic as a catalyst for “Digital sustainable development” and the “Digital transformation” of university teaching have achieved substantial academic impact (150 citations), reflecting a pressing concern with the temporal sustainability of emergency remote teaching models.

Thirdly, the discourse is heavily shaped by the integration of mobile and immersive technologies as active mediators of educational timespaces. Empirical and quasi-experimental studies frequently focus on how emerging digital tools construct localised, immersive learning environments. For example, research employing “Spherical video-based virtual reality (SVVR)” to support descriptive writing in high schools (127 citations) and studies evaluating “Immersive virtual reality (VR)” in inquiry-based physics (58 citations) demonstrate a thematic trend toward utilizing technology to overcome physical distance and alter cognitive load and experiential learning. On a more ubiquitous level, phenomenological research exploring students’ “Lived experiences” with “Smartphones” across “diverse learning contexts” (77 citations) and case studies on “Collaborative reading” through “Social annotation” (73 citations) illustrate how mobile devices and web-based platforms dynamically mediate student engagement and peer connection across fluid, asynchronous learning spaces.

Finally, the discourse is underpinned by a robust quantitative trend that examines the psychosocial and cultural realities of learners navigating these digital spaces. The most highly cited study in the corpus (243 citations) utilises Structural Equation Modelling (SEM) and Partial Least Squares (PLS) to position psychological traits—specifically “Grit,” “Perseverance,” and “Stamina”—as critical “Success determinants” within e-learning systems. Similarly, quantitative investigations into the “Cultural impacts on e-learning systems’ success” (156 citations) reveal that dimensions such as “Culture” and “Individualism/collectivism” significantly influence student satisfaction and system use across higher education institutions. This indicates that the academic conversation regarding digital educational space is not merely infrastructural but deeply intertwined with the cultural and psychological capacities of the students inhabiting those environments.

### 5.5 Cluster structural observations and gap concerns

The overall network and cluster structure of digital learning and educational chronotope scholarship, created cumulatively, resembles a multi-nodal map, with topic clusters positioned around its centre and somewhat hollow, and less densely researched at the intersections between theoretical pedagogy and systemic application ( Figure 3). This structure is particularly evident in the analysis of thematic density ( Figure 4), which makes the map easier to comprehend because of the lack of substantial interrelationships and overlaps among certain methodological clusters. On the other hand, the distinct separation visible in both the density and overlay maps ( Figures 4 and 5) provides striking evidence of the field’s significant fragmentation into disconnected subfields.

**Figure 3.**
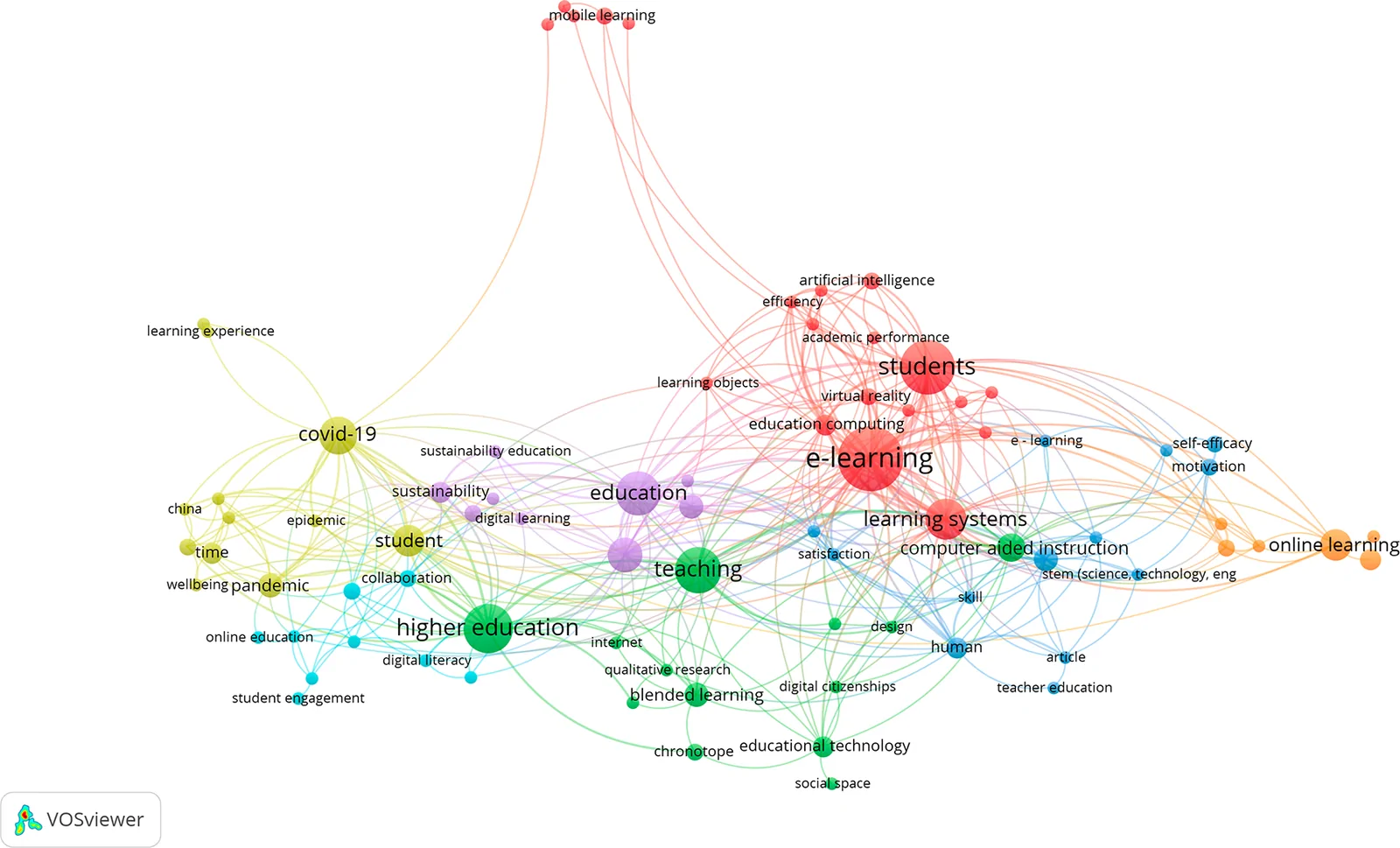
Network diagram of key co-occurrences. Source: Own elaboration.

**Figure 4.**
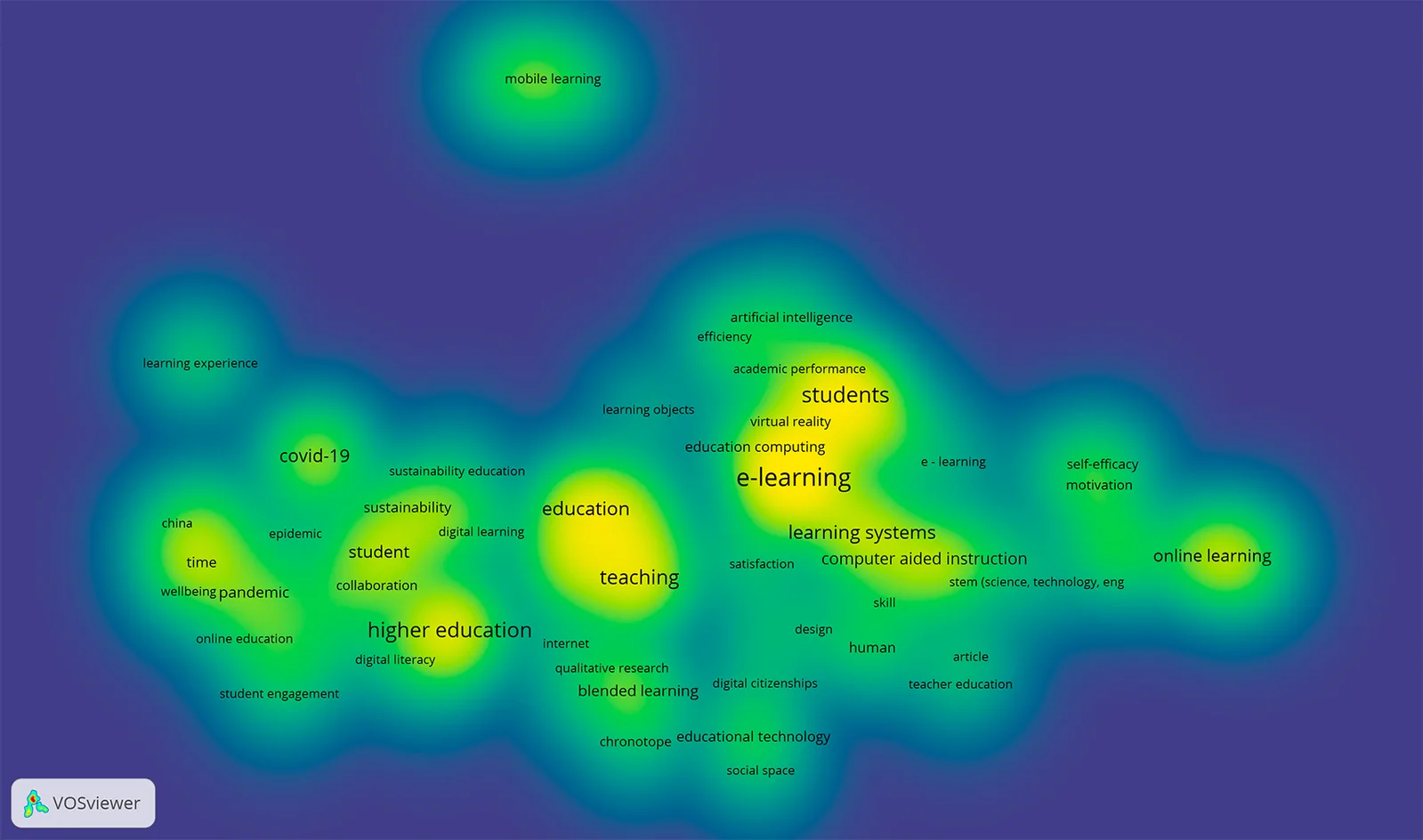
Density Visualisation of Key Co-occurrences. ource: Own elaboration.

**Figure 5.**
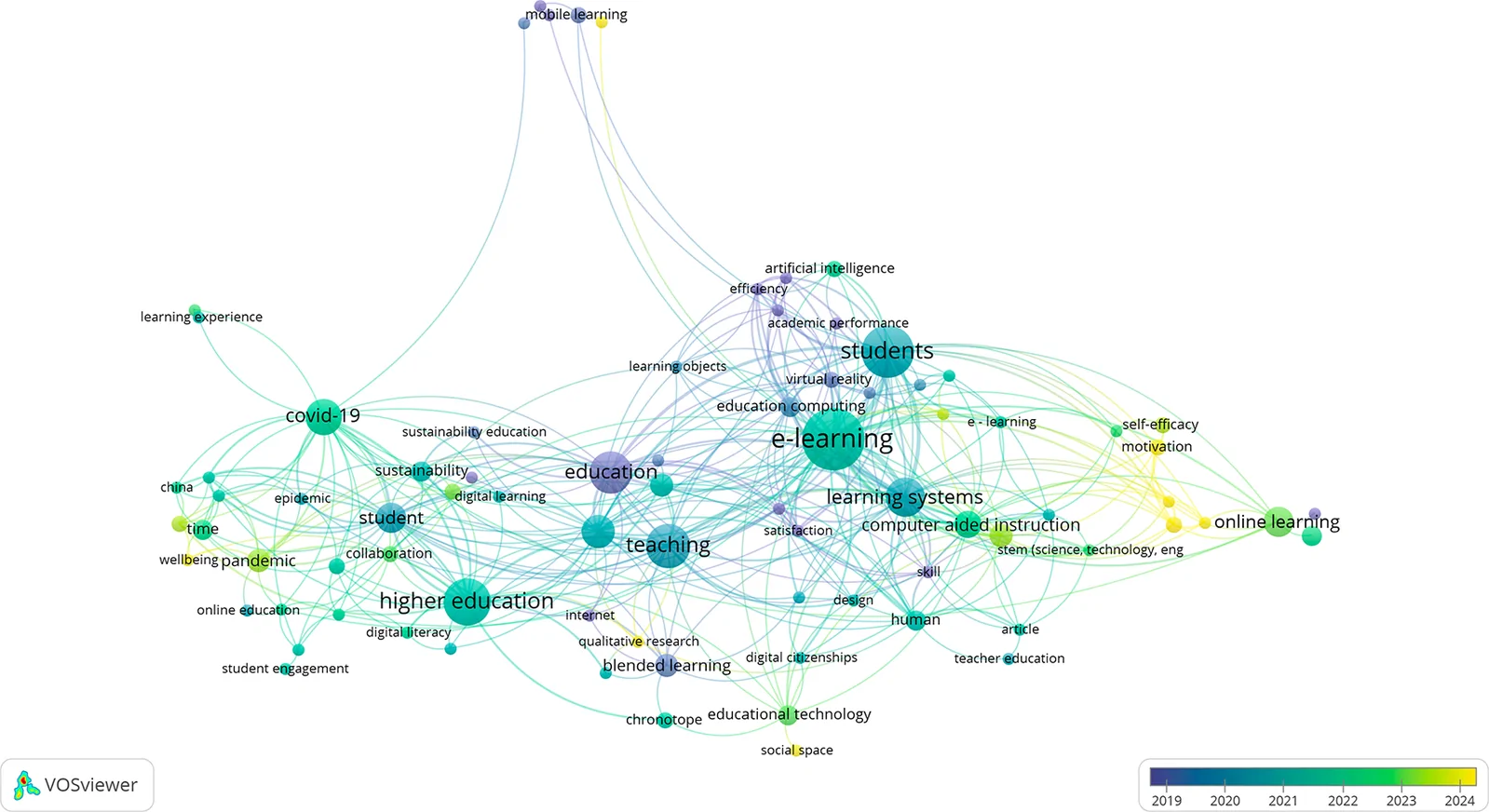
Overlay visualisation of Key Co-occurrences. Source: Own elaboration.

The most notable observation of this study is that Cluster 4: Pandemic-Induced Educational Chronotopes and Student Mobility has been isolated in its temporal reactivity. The distance between this cluster and others reveals that research on emergency remote teaching and the chronotope is considerably distant from other research fields. This scholarship examines how the global health crisis altered the lived realities of mobile learners and generated asynchronous, precarious temporalities that affected student well-being. These practices appear to be disconnected from a deeper exploration of long-term, sustainable learning approaches, as this cluster is separated from Cluster 2, Chronotopic Design of Blended Learning in Higher Education. Within critical pedagogy, optimising psychological factors such as self-efficacy and motivation is crucial not only for academic performance but also for student agency and mutual knowledge-building in digital spaces. Teacher development should go beyond technical skills to include critical capabilities for the ethical design of hybrid environments, ensuring that digital tools challenge pedagogical hierarchies. Aligning these psychosocial factors and literacies with the goals of equity and dialogue enables institutions to move from reactive measures to sustainable educational ecologies that reduce spatiotemporal insecurities and support student voice.

Additionally, this study observes that AI-Driven E-Learning Systems (Cluster 1) and Psychological Determinants of E-Learning in STEM Education (Cluster 3) form a macro-cluster, demonstrating that research on the extent to which learners adopt technology is deeply intertwined with cognitive and affective evaluations. Due to their interconnected nature, the functions of highly personalised learning environments driven by educational computing and the quantitative measurement of student motivation, self-efficacy, and satisfaction constitute a macro-cluster. Scholars are heavily invested in this field of study to map the complex pathways between technology acceptance and academic performance. In other words, while the vast majority of articles in this macro-cluster examine the determinants of e-learning success and user satisfaction, they remain structurally separated from the critical, qualitative perspectives of postdigital pedagogies.

### 5.6 Responding to the study questions and proposals for future research

The conceptual and thematic landscape shaping the discourse on space and time in digital higher education is characterised by four primary trajectories. First, there is a structural reconceptualisation of learning environments that deliberately dissolves the rigid dichotomies (i.e., grey space) between physical and virtual spaces, advancing instead toward integrated, post-digital educational ecologies. Second, the discourse is heavily influenced by systemic disruptions, most notably the COVID-19 pandemic, which foregrounded issues of international student immobility, infrastructural adaptation, and the urgent need for digital development. Third, mobile and immersive technologies, such as spherical video-based virtual reality and smartphones, have emerged as an asynchronous learning solution to overcome physical distance. Finally, a robust quantitative thematic trend underscores that navigating these digital environments is deeply intertwined with learners’ psychosocial and cultural capacities, positioning psychological traits such as grit and perseverance, as well as cultural background, as critical determinants of e-learning success.

Critical pedagogy intersects with chronotopic perspectives by exposing the deeply political nature of educational space and time. This demands the conscious redesign of these dimensions to foster equity, student agency, and social interaction. This intersection is highly evident in the drive to democratise knowledge through digital learning environments, which function as active, connective spaces that blur traditional pedagogical hierarchies and validate student voice. Furthermore, critical pedagogy necessitates the design of equitable digital chronotopes to actively mitigate the uncertainty that physically grounded transnational students face and that forces them into precarious, asynchronous liminal spaces. By purposefully aligning technological affordances with constructivist pedagogical principles, educators can transcend treating technology as merely a passive information-delivery system.

An analysis of the current scholarship’s structural network reveals significant fragmentation, highlighting key research gaps and emerging themes for future inquiry. A primary gap exists in the reactivity of pandemic-focused research. Consequently, future research must react to lessons from global disruptions in the deliberate, long-term design of resilient and equitable frameworks. The bibliometric analysis indicates that studies on AI-driven e-learning systems and psychological factors in education constitute a prominent and tightly connected quantitative research cluster. Despite this strong internal cohesion, it shows limited engagement with critical and qualitative research that examines the broader social, pedagogical, and ethical implications of digital technologies in education. Addressing the emerging themes and structural research gaps identified in the bibliometric mapping (RQ3), the following three areas are recommended for future scholarly inquiry:

Bridging Emergency Past Decisions and Sustainable Design: The network analysis revealed a structural isolation between reactive studies on pandemic-induced emergency remote teaching and the proactive chronotopic design of blended learning. Future research must bridge this gap by investigating how lessons from involuntary disruption can inform the deliberate, long-term redesign of digital learning environments that remain resilient amid future global disruptions. ^25^ ^,^ ^22^

Integrating AI-Driven E-Learning with Critical Postdigital Pedagogies: A significant macro-cluster centres on the quantitative measurement of AI-driven e-learning success and its psychological determinants, yet it remains structurally separated from pedagogies. Future mixed-methods research should explore how highly personalised, data-driven learning environments can be optimally designed not only to maximise algorithmic efficiency but also to mitigate frustration with need and foster dialogue. ^35^ ^,^ ^47^

Optimising Psychological Determinants and Teacher Competencies: Building on the psychological focus of e-learning in STEM, further investigation is required into how specific immersive tools (e.g., VR and educational games) can be designed to directly boost intrinsic motivation and self-efficacy. Future research should explore how game-based learning environments can be ethically designed to reduce time and space constraints and promote a stronger sense of belonging in postdigital learning environments.

## 6. Conclusion

Digital learning offers a transformative postdigital opportunity for higher education, but its potential depends on how institutions address persistent structural and conceptual gaps. This study’s bibliometric review (2015–2025) highlights a multi-nodal research landscape and underscores the need for more coherent and sustained inquiry into the spatial and temporal dimensions of digital learning. It argues that technology should be understood as a mediating force that requires careful alignment with constructivist pedagogy, ongoing professional development, and distributed leadership to support equitable and flexible learning environments. At the same time, meaningful digital education must prioritise the human experience by fostering student well-being, a sense of belonging, and active participation in increasingly complex learning contexts.

## Declaration of generative AI and AI-assisted technologies in the writing process

While preparing this work, the authors used ChatGPT and Grammarly to improve the quality of their writing. After using these tools, the author reviewed and edited the content as needed and took full responsibility for the publication’s content.

## Data Availability

The datasets generated and analysed during the current study are available in the Figshare repository: The PRISMA checklist is available on Figshare at 10.6084/m9.figshare.32984147 under a CC0 license. ^58^ The values used to build graphs or other figures are available in the Dataset for Educational Chronotopes in the Digital Landscape of Higher Education: A Systematic Bibliometric Review of Research Trends, 2015–2025, available at Figshare: 10.6084/m9.figshare.32984450 under a CC-BY 4.0 license. ^59^ The PRISMA 2020 flow diagram used to document the study selection process for *Educational Chronotopes in the Digital Landscape of Higher Education: A Systematic Bibliometric Review (2015–2025)* is available in the Figshare repository at https://doi.org/10.6084/m9.figshare.33005486 under a Creative Commons Attribution 4.0 International (CC BY 4.0) license. ^60^
