# Supplementary material for: Educational Chronotopes in the Digital Landscape of Higher Education: A Systematic Bibliometric Mapping of Research Trends, 2015–2025
Source: F1000Res. 2026 Aug 6;15:1309. [Version 1] doi: 10.12688/f1000research.186901.1 (PMC13770167; doi:10.12688/f1000research.186901.1)
Supplement: Peer review history

# Reviewer report: Reviewer response for version 1

**DOI:** 10.5256/f1000research.206280.r523006

**Published:** 2026-11-14

**Authors:** Godson Nwokocha

**Affiliations:** 1 STADIO Higher Education, Cape Town, South Africa

This is a relevant and well-structured review that makes a useful contribution to understanding research on educational chronotopes in digital higher education. The research questions, methodology, analysis and conclusions are generally well aligned. The findings offer useful insights into existing research trends, emerging areas of interest and gaps that may inform future scholarship and practice. I am satisfied that the manuscript meets the requirements for publication and recommend acceptance.

 The rationale for the study is clearly presented. The authors identify a relevant gap in the literature concerning how educational chronotopes (the relationship between time and space) are understood and explored in digital higher education. The study's objectives are appropriately aligned with this gap and are further clarified through three research questions. These questions provide a clear direction for the review and establish what the authors intend to investigate, particularly in relation to emerging themes, critical pedagogy and gaps in existing research.

 The methodology is described in sufficient detail to allow readers to understand how the review was conducted. The authors explain the database used (Scopus), the search strategy, the selection criteria and the screening process. The use of PRISMA 2020 further supports transparency in the identification and selection of relevant studies. The final sample of 112 publications is clearly reported. In addition, the authors explain the bibliometric techniques used, including citation analysis, bibliographic coupling, co-citation analysis and keyword co-occurrence, as well as the use of VOSviewer to generate and interpret the visualisations. Overall, the methodological approach is appropriate and sufficiently transparent.

 The bibliometric techniques are suitable for the purpose of mapping research trends, relationships and thematic developments within the field. The authors make appropriate use of citation patterns, keyword networks and visual representations to identify connections between publications and research themes. The interpretation of these results is generally clear and contributes to answering the research questions.

 The conclusions are generally consistent with the findings presented. The discussion draws together the main research clusters and highlights the fragmented nature of scholarship on educational chronotopes in digital higher education. The authors also identify relevant implications for critical pedagogy, student agency, equity and the design of digital learning environments. The limitations of the study and the potential constraints associated with the search strategy and selected database are acknowledged. This provides an appropriate basis for interpreting the findings and considering their applicability.

 The review's approach is appropriate to its stated scope. The article does not present itself as a Living Systematic Review; it reports a bibliometric mapping based on a defined search and selection process. An ongoing search schedule is therefore not required for the type of review undertaken.

Are the rationale for, and objectives of, the Systematic Review clearly stated?

Yes

Is the statistical analysis and its interpretation appropriate?

Yes

If this is a Living Systematic Review, is the ‘living’ method appropriate and is the search schedule clearly defined and justified? (‘Living Systematic Review’ or a variation of this term should be included in the title.)

Yes

Are sufficient details of the methods and analysis provided to allow replication by others?

Yes

Are the conclusions drawn adequately supported by the results presented in the review?

Yes

Reviewer Expertise:

NA

I confirm that I have read this submission and believe that I have an appropriate level of expertise to confirm that it is of an acceptable scientific standard.

# Reviewer report: Reviewer response for version 1

**DOI:** 10.5256/f1000research.206280.r520026

**Published:** 2026-10-24

**Authors:** Rochelle Wessels

**Affiliations:** 1 University of South Africa, Pretoria, South Africa

**Educational Chronotopes in the Digital Landscape of Higher Education: A Systematic Bibliometric Mapping of Research Trends, 2015–2025**

 The rationale for conducting the systematic review and the objectives of the article are clearly articulated. The research methodology is appropriately described, and the discussion of the findings aligns with the stated objectives of the study.

 The article investigates the expansion of digital learning in higher education and its influence on the spatial and temporal dimensions, or chronotopes, of teaching and learning. A quantitative bibliometric research design (2015-2025) was employed to analyse publication trends, using citation analysis and keyword co-occurrence to identify key thematic clusters and influential publications.

 This article employs Bakhtin's construct of the chronotope to analyse the complex interactions, which refers to the “intrinsic connectedness of temporal and spatial relationships”. Within digital environments, space is understood as a social, cultural, and relational construct that facilitates dialogue, whereas time encompasses the pace, rhythms, and sequencing of educational activities. While Bakhtin’s chronotope concept provides a theoretical basis for exploring the interrelationship between time and space in educational contexts, its use within the education field remains dispersed and insufficiently integrated.

 Guided by the PRISMA 2020 guidelines, this review explores how existing research has applied the concept of the chronotope to understand digital learning within higher education and teacher education. The review adopted a structured approach to identifying, selecting, and analysing relevant literature to support methodological transparency, consistency, and reproducibility.

 The initial search of the Scopus database identified 401 records from 2015 to 2025. Following the application of the inclusion and exclusion criteria, 112 articles were deemed eligible and retained for the final analysis.

 The thematic coupling findings highlight five major domains in which critical pedagogy converges with emerging digital learning approaches. These connections are evident in efforts to intentionally reshape educational spaces and time structures in ways that advance equity, learner autonomy, and meaningful social interaction. The dialogical perspective of the chronotope serves as an analytical lens for exploring how spatial and temporal relationships are socially formed, interconnected, and continually negotiated by participants engaged in collaborative learning.

 Digital learning provides considerable opportunities to transform higher education, although its full potential can only be realised by addressing persistent structural and conceptual challenges. The bibliometric findings highlight the importance of conducting more cohesive and sustained research into the spatial and temporal dimensions of digital learning. The study further underscores the role of technology as a mediating mechanism in developing inclusive and flexible educational environments. Moreover, effective digital education should prioritise the human dimension of learning by supporting student well-being, fostering a sense of belonging, and promoting active and meaningful participation.

Are the rationale for, and objectives of, the Systematic Review clearly stated?

Yes

Is the statistical analysis and its interpretation appropriate?

Yes

If this is a Living Systematic Review, is the ‘living’ method appropriate and is the search schedule clearly defined and justified? (‘Living Systematic Review’ or a variation of this term should be included in the title.)

Not applicable

Are sufficient details of the methods and analysis provided to allow replication by others?

Yes

Are the conclusions drawn adequately supported by the results presented in the review?

Yes

Reviewer Expertise:

Public Administration and Management

I confirm that I have read this submission and believe that I have an appropriate level of expertise to confirm that it is of an acceptable scientific standard.
